# Heteronemin, a marine natural product, sensitizes acute myeloid leukemia cells towards cytarabine chemotherapy by regulating farnesylation of Ras

**DOI:** 10.18632/oncotarget.24771

**Published:** 2018-04-06

**Authors:** Minakshi Saikia, Archana P Retnakumari, Shabna Anwar, Nikhil P Anto, Rashmi Mittal, Shabna Shah, Kavya S Pillai, Vinod S Balachandran, Vidya Peter, Reeba Thomas, Ruby John Anto

**Affiliations:** ^1^ Division of Cancer Research, Rajiv Gandhi Centre for Biotechnology, Thiruvananthapuram, Kerala, India; ^2^ Department of Biotechnology, Maharishi Markandeshwar University, Haryana, India; ^3^ Research Scholar, University of Kerala, India

**Keywords:** acute myeloid leukemia, Ras, heteronemin, farnesyl transferase, chemosensitization

## Abstract

Cytarabine is a conventionally used chemotherapeutic agent for treating acute myeloid leukemia (AML). However, chemoresistance, toxic side-effects and poor patient survival rates retard the efficacy of its performance. The current study deals with the chemosensitization of AML cells using heteronemin, a marine natural product towards cytarabine chemotherapy. Heteronemin could effectively sensitize HL-60 cells towards sub-toxic concentration of cytarabine resulting in synergistic toxicity as demonstrated by MTT assay and [^3^H] thymidine incorporation studies, while being safe towards healthy blood cells. Flow cytometry for Annexin-V/PI and immunoblotting for caspase cleavage proved that the combination induces enhancement in apoptosis. Heteronemin being a farnesyl transferase inhibitor (FTI) suppressed cytarabine-induced, farnesyl transferase-mediated activation of Ras, as assessed by Ras pull-down assay. Upon pre-treating cells with a commercial FTI, L-744,832, the synergism was completely lost in the combination, confirming the farnesyl transferase inhibitory activity of heteronemin as assessed by thymidine incorporation assay. Heteronemin effectively down-regulated cytarabine-induced activation of MAPK, AP-1, NF-κB and c-myc, the down-stream targets of Ras signaling, which again validated the role of Ras in regulating the synergism. Hence we believe that the efficacy of cytarabine chemotherapy can be improved to a significant extent by combining sub-toxic concentrations of cytarabine and heteronemin.

## INTRODUCTION

Acute Myeloid Leukemia (AML) is a hematological malignancy implicated by the abnormal clonal proliferation of myeloid progenitor cells in bone marrow and peripheral blood. Immature blasts accumulate in the bone marrow and the congregation of these immature myeloid blasts ultimately results in conditions such as granulocytopenia, thrombocytopenia, and/or anemia [1]. The frontline intensive chemotherapy for AML is “7 + 3” induction regimen using cytarabine (Ara-C) and daunorubicin or idarubicin [2]. Unlike other cancer types, AML is extremely heterogeneous, associated with a multitude of molecular abnormalities [3, 4]. High relapse rates (~70%) along with chemoresistance and side effects of the drug negatively affect the disease free survival of the patients with AML [5]. This necessitates an urgent call for novel therapeutic regimens for AML [6].

Studies conducted over the past few years have unveiled the molecular mechanisms behind disease progression, relapse and drug resistance of AML. The oncogenic Ras mutations and the mutations in other components of Ras/MAPK signaling pathways appear to be mutually exclusive events in most tumors, indicating that deregulation of Ras-dependent signaling is a critical requirement for tumorigenesis. Ras proteins are small GTPases that play a pivotal role in signal transduction and eventually regulate cell proliferation, survival, and differentiation. ~30% of all human cancers have been reported to have constitutive activation of Ras [7]. The three major isoforms of Ras oncogenes include *KRAS, NRAS* and *HRAS* and different Ras oncogenes are preferentially associated with different types of human cancer [8]. Several studies have indicated that, gain of function mutation causes the transformation of Ras from a proto-oncogene to an oncogene, leading to its constitutive activation. Apart from this, over-expression of Ras protein is sufficient to confer a transforming potential to the cells, which points to the significance of Ras in carcinogenesis [9]. In AML also, Ras activation is a major trigger for the onset of leukemiogenesis as well as its progression [10]. To be biologically active, Ras translocates from cytoplasm to plasma membrane aided by several post-translational modifications. Addition of a farnesyl group to the Ras C-terminal cysteine aided by farnesyl tranferase enzyme is one of the prominent post-translational modifications. Currently, several compounds have been developed which are known to inhibit tumor development by inhibiting farnesylation [11].

In the current study, we have used heteronemin, a sesterterpene isolated from marine sponges, for sensitizing AML cells towards the action of cytarabine. Heteronemin is reported to exhibit anti-tumor and anti-microbial properties [12–15]. It is also reported to be a modulator of TNFα-induced NF-κB pathway [16]. Ledroit *et al.* has reported that heteronemin exhibits farnesyl transferase inhibitory action [17]. Since farnesylation and subsequent activation of Ras, induced by cytarabine is crucial in determining the response of leukemic cells towards cytotoxic action of cytarabine, we presumed that heteronemin can act as an effective chemosensitizer for cytarabine chemotherapy.

## RESULTS

### Cytarabine and heteronemin exerts synergistic cytotoxic effect in acute myeloid cells HL-60, while being safe for the peripheral blood mononuclear cells (PBMCs)

**Figure 1:**
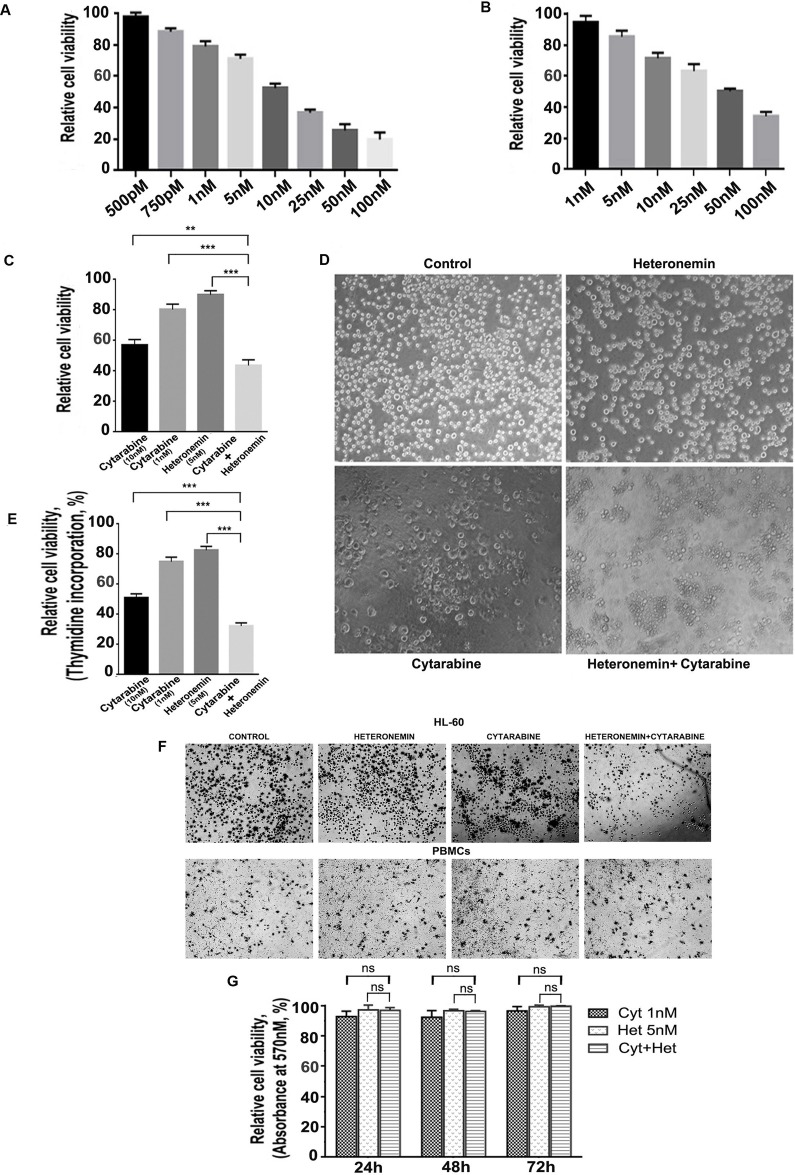
Heteronemin sensitizes HL-60 cells towards Cytarabine exerting synergistic cytotoxic effect without affecting healthy blood cells (**A**) Relative viability of HL-60 cells treated with different concentrations of cytarabine (500 pM to 100 nM) for 72 h as assessed by MTT assay (**B**) Relative viability of HL-60 cells treated with different concentrations of heteronemin (1 nM to 100 nM) for 72 h as assessed by MTT assay (**C**) MTT assay showing the relative cell viability of HL-60 cells treated with cytarabine (10 nM) and (1 nm) individually ,Heteronemin (5 nM) and a combination of 1 nM cytarabine and 5 nM heteronemin. Cells were pre-treated with 5 nM heteronemin for 6 h prior to the treatment of cytarabine (1 nM). The cell viability was assessed by MTT assay after 72 h of cytarabine addition (**D**) Morphological changes induced by the individual compounds and combination of 1 nM cytarabine and 5 nM heteronemin were compared with control HL-60 cells. Cells were treated with the combination and were photomicrographed, before performing MTT assay (**E**) Thymidine incorporation assay showing the synergistic cytotoxic effect of 1 nM cytarabine and 5 nM heteronemin in HL-60 cells (**F**) Photomicrographs showing formazan crystal formation in HL-60 cells (upper panel) and healthy PBMCs (lower panel) upon treating the cells with a combination of 1 nM cytarabine and 5 nM heteronemin as described before (**G**) Relative viability of PBMCs treated with a combination of 1 nM cytarabine and 5 nM heteronemin as assessed by MTT assay Cells were pre-treated with 5 nM heteronemin for 6 h and further treated with 1 nM cytarabine for different time intervals (24 h, 48 h and 72 h) and cell viability was calculated.

Cell viability assay was conducted to assess the cytotoxic effect of cytarabine and heteronemin in the AML cell line, HL-60 (M3 subtype of AML according to the FAB classification). Both the compounds showed dose-dependent cytotoxicity as shown in Figure 1A, 1B. Our next attempt was to identify a sub-toxic concentration of cytarabine, which when combined with heteronemin, can induce a synergistic toxicity at dose well below the IC 50 dose of cytarabine alone. With this purpose we tried different combinations of these compounds, varying the pre-treatment time of heteronemin from 1 h to 8 h (data not shown) and conducted MTT assay. A combination of 1 nM cytarabine in HL-60 cells, pre-treated for 6 h with 5 nM heteronemin produced 56% cytotoxicity, which was significantly higher than that produced by either cytarabine (~20%) or heteronemin (~10%) alone (Figure 1C). Photomicrograph of various wells also supported this observation (Figure 1D). Further, we confirmed the synergistic toxicity using [^3^H] thymidine incorporation assay, where 1 nM cytarabine with 5 nM heteronemin produced 67% cytotoxicity, which was higher than that of either cytarabine (~25%) or heteronemin (~18%) alone (Figure 1E). In both cases, the synergism was significantly high and the CI was less than 1 as assessed by the method of Chou and Talaly [21]. The remarkable observation in the study was that the cytotoxicity produced by the combination of 1 nM cytarabine and 5 nM heteronemin was more than that produced by even a 10 times higher dose of cytarabine (10 nM) (Table 1). Interestingly, this combination did not affect the viability of peripheral blood mononuclear cells (PBMCs) isolated from healthy donors, which was evident from the pattern of formazan crystal formation. This data together with the results from MTT assay confirmed its biological safety in healthy blood cells (Figure 1F, 1G). These observations indicate that, a therapeutic benefit can be eventually achieved using a sub-toxic concentration of cytarabine, when used in combination with heteronemin, which will not affect the viability of healthy blood cells.

**Table 1:** Heteronemin induces synergistic cytotoxicity in HL-60 cells

HL-60 Cells	% of cytotoxicity				
	Cyt (10 nM)	Cyt (1 nM) c	Het (5 nM) h	Cyt+Het *n*	Synergism S = *n*-(c+h)
MTT assay	43	20	10	56	26
[^3^H] Thymidine assay	49	25	18	67	24

### A combination of cytarabine and heteronemin induces synergistic enhancement in membrane flip-flop, nuclear membrane damage and apoptosis in HL-60 cells

**Figure 2:**
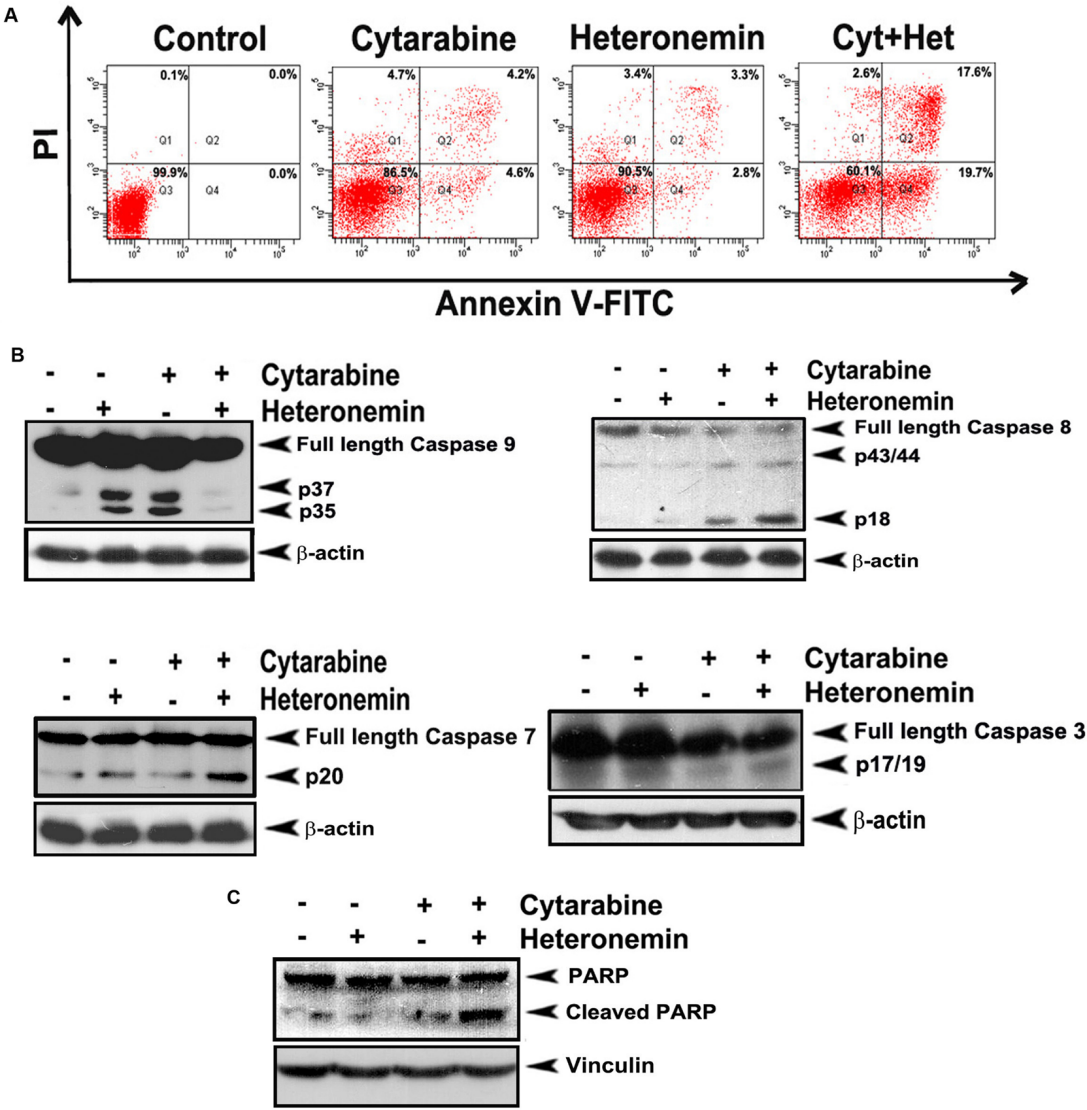
Heteronemin pre-treatment enhances apoptosis induced by cytarabine in HL-60 cells (**A**) Flow cytogram showing Annexin V/PI staining of cells treated with the combination of 1 nM cytarabine and 5 nM heteronemin. Cells were treated with 5 nM heteronemin for 6 h and later with 1 nM cytarabine, stained with fluorescein isothiocyanate (FITC)-conjugated Annexin V and propidium iodide and subjected to flow cytometry. The population of Annexin/PI-positive cells in the top right and bottom right quadrants represent the total percentage of apoptotic cells (**B**) Western blots showing caspase activation in HL-60 cells treated with the combination. Whole-cell lysates (WCL) were prepared after treating HL-60 cells with the drugs as described earlier and were resolved on a 15% gel and subjected to Western blotting using antibodies against the caspases 9, 8, 7, 3 and were detected by ECL (**C**) Western blot showing PARP cleavage in cells treated with a combination of 1 nM cyatarabine and 5 nM heteronemin as described before. After 48 h whole cell lysate was resolved on an 8% gel, immunoblotted against anti-PARP and detected by ECL. β-actin and vinculin were used as loading controls.

Next we analyzed the mode of cell death induced by the combination of cytarabine and heteronemin in HL60 cells. Flow cytometry was performed to study whether membrane flip-flop and loss in nuclear membrane integrity, which is indicative of programmed cell death, is induced by the compounds, individually or in combination. The results showed that a significant enhancement in both early (~19.7%) and late stages of apoptosis (~17.6%) is exhibited by the cells pre-treated with heteronemin prior to cytarabine treatment as compared to either cytarabine alone (early apoptosis ~4.6%, late apoptosis ~4.2%) or heteronemin alone (early apoptosis ~2.8%, late apoptosis ~3.3%) (Figure 2A). Western blotting showed significant cleavage of procaspase 9, procaspase 8, procaspase 7 and procaspase 3 (Figure 2B) to their respective active fragments, indicating programmed cell death in the leukemic cells treated with heteronemin and cyatarabine. In the case of caspase 9, we found that the synergistic combination of cytarabine and heteronemin induced significant degradation of even the cleaved fragment. A remarkable cleavage of PARP, the downstream target of caspase cascade, was also observed upon combination treatment (Figure 2C), confirming that heteronemin is chemosensitizing the leukemic cells towards cytarabine through enhancement of apoptosis.

### Heteronemin significantly inhibits the activation of Ras, induced by cytarabine in HL-60 cells, leading to a synergistic inhibition of DNA synthesis, which is lost when pre-treated with an inhibitor of farnesyl transferase, an enzyme necessary for Ras activation

**Figure 3:**
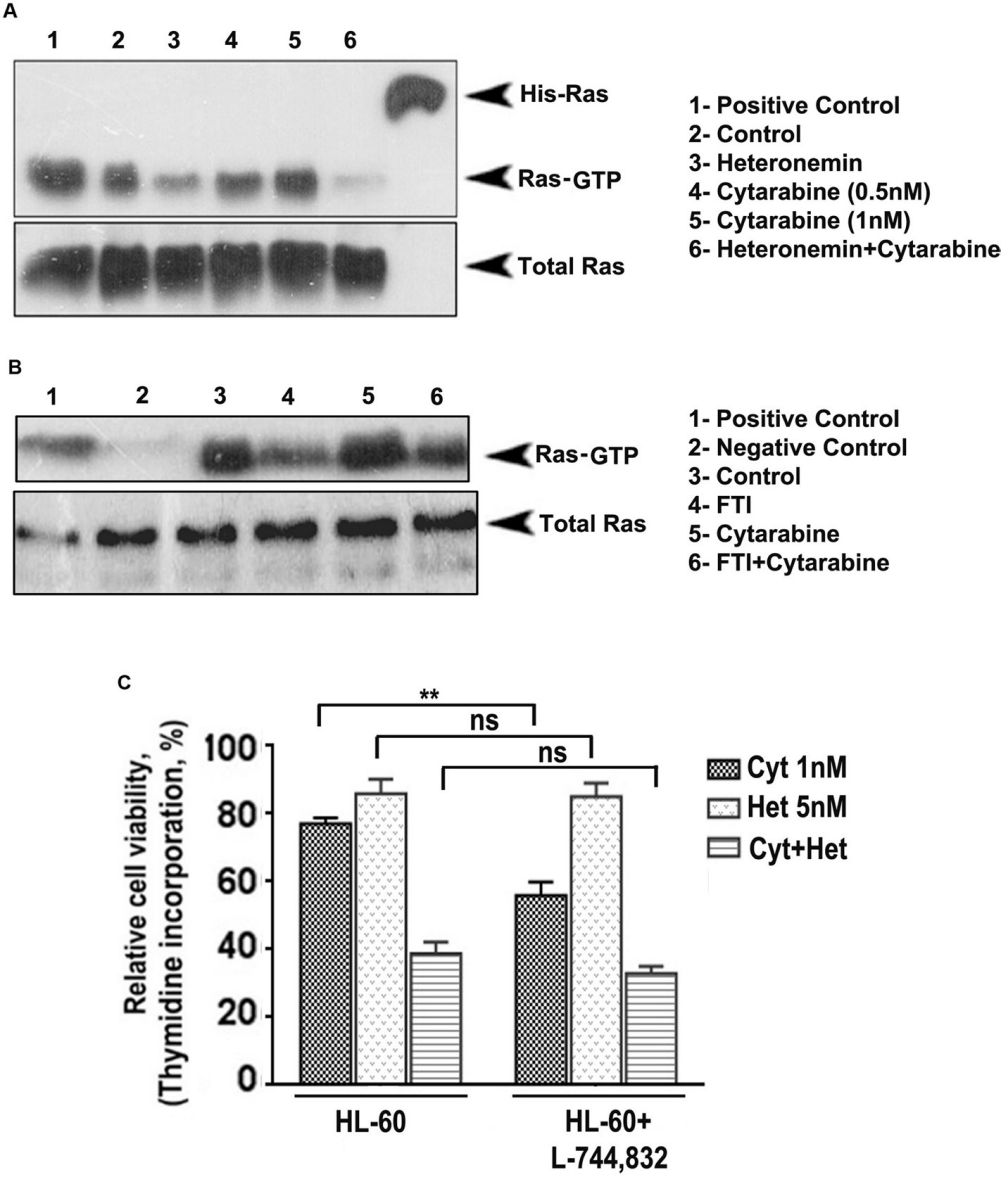
Heteronemin pre-treatment suppresses activation of Ras by inhibiting farnesyl transferase enzyme (**A**) Ras pull down assay showing suppression in cytarabine-mediated Ras-GTP formation in HL-60 cells pre-treated with heteronemin. HL-60 cells were pre-treated with 5 nM heteronemin and later with 1 nM cytarabine. Ras pull down assay was performed in the cell lysates according to manufacturer's instructions. (**B**) Ras pull down assay conducted in HL-60 cells using a combination of 5 μM L-744,832, a commercially available farnesyl transferase inhibitor, and 1 nM cytarabine (**C**) Thymidine incorporation assay following 1 h pre-treatment of L-744,832, in HL-60 cells indicating no significant difference between HL60 set and HL60+ L-744,832 set for single agent heteronemin or heteronemin + cytarabine since both L-744,832 and heteronemin targets farnesyl transferase for inhibition.

Activated Ras plays a pivotal role in regulating the growth and proliferation of cells. For Ras to get activated (Ras-GTP formation), Ras undergoes several post-translational modifications, which in turn facilitate its attachment to the inner surface of the plasma membrane. One of the most critical modifications is farnesylation i.e. the addition of a farnesyl isoprenoid moiety and the reaction is catalyzed by the enzyme farnesyl transferase (FTase). For the past few years, clinical application of farnesyl transferase inhibitors (FTIs) has gained considerable interest. Preclinical studies have shown that FTIs cause tumor regression by both apoptosis and cell cycle regulation. Ledroit *et al*. has reported that heteronemin is a potential farnesyl transferase inhibitor [17] and hence, we strongly presume that it might successfully inhibit farnesylation and subsequent activation of Ras. We conducted a Ras pull-down assay to check whether heteronemin causes an inhibiton of cytarabine-induced Ras activation and whether it has any role in regulating heteronemin's chemosensitization towards cytarabine. It was very interesting to see that, in contrast to the activation of Ras-GTP in the cytarabine-treated cells, the cells pre-treated with heteronemin, exhibited a drastic reduction in the expression of active Ras (Figure 3A). In order to confirm the role of Ras in regulating the synergism, we treated the cells with L-744,832, a commercially used farnesyl transferase inhibitor, either alone or in combination with cytarabine and conducted another pull-down assay. The results illustrated a significant reduction in the Ras expression, upon inhibition of farnesyl transferase, attesting the pivotal role of Ras in regulating the synergism (Figure 3B). To confirm this inference, we conducted [^3^H] thymidine incorporation assay and checked whether pre-treatment with L-744,832 affects the synergistic cytotoxic effect of the combination of heteronemin and cytarabine. When cytarabine was added to cells pre-treated with L-744,832, there was a reduction in cell viability. However, since a sub-toxic concentration of L-744,832 was used, there was no significant decrease in viability in the case of L-744,832 heteronemin combination, although both of them are targeting farnesyl transferase. Since both L-744,832 and heteronemin targets farnesyl transferase for inhibition, there is no significant difference between HL60 set and HL60+ L-744,832 set for single agent heteronemin or heteronemin+cytarabine (Figure 3C). These results illustrate that inhibition of farnesylation of Ras by heteronemin has a key role in regulating its chemosensitizing efficacy in cytarabine chemotherapy of leukemic cells.

### Pre-treatment with heteronemin inhibits cytarabine-mediated activation of various pathways instigated by Ras signaling

**Figure 4:**
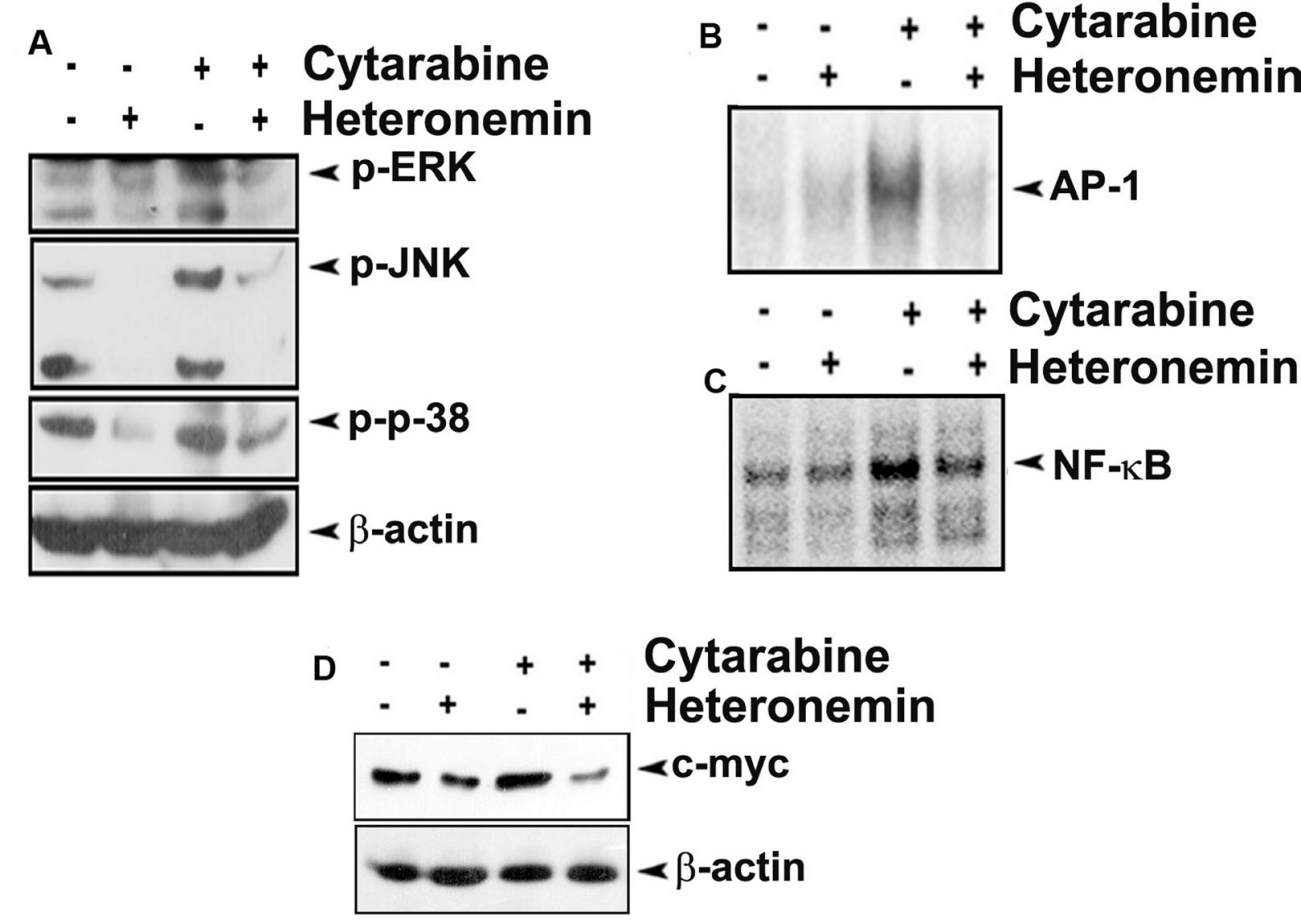
Heteronemin pre-treatment inhibits cytarabine mediated activation of MAPK signaling in HL-60 cells (**A**) Heteronemin down-regulates cytarabine-mediated activation of ERK, JNK and p-38 in HL-60 cells. HL-60 cells were pre-treated with 5 nM heteronemin for 6 h, prior to treatment with 1 nM cytarabine. Whole cell lysate was resolved in 10% gel and immunoblotted against antibodies specific against phosphorylated ERK, JNK and p-38 (**B**) Heteronemin pre-treatment causes reduction in cytarabine mediated nuclear translocation of AP-1. HL-60 cells were treated with 5 nM heteronemin for 6 h, prior to treatment with 1 nM cytarabine. Nuclear extracts were prepared and EMSA was performed to assess the nuclear translocation of AP-1 transcription factor (**C**) Heteronemin pre-treatment causes reduction in cytarabine mediated nuclear translocation of NF-κB. HL-60 cells were treated with 5 nM heteronemin for 6 h, prior to treatment with 1 nM cytarabine. Nuclear extracts were prepared and EMSA was performed to assess the nuclear translocation of NF-κB transcription factor (**D**) Heteronemin pre-treatment resulted in down-regulation of cytarabine-mediated expression of c-myc. HL-60 cells were pre-treated with 5 nM heteronemin for 6 h, prior to treatment with 1 nM cytarabine. Whole cell lysate was resolved in 10% gel and immunoblotted against c-myc antibody.

We also checked the effect of the compounds individually and in combination, in regulating the down-stream signaling pathways, which are regulated by Ras during the progression of AML. First we looked for the members of the MAPK pathway, which is one of the prominent downstream targets of Ras signaling. As speculated, pre-treatment with heteronemin drastically down-regulated cytarabine-mediated phosphorylation of ERK, JNK and p38, the key molecules involved in MAPK signaling (Figure 4A). Moreover, cytarabine-induced nuclear translocation of AP-1, the downstream effector molecule of MAPK pathway, was almost completely inhibited by heteronemin as assessed by EMSA (Figure 4B).

The studies done by Norris *et al.* have demonstrated that NF-κB is a major pathway activated by oncogenic Ras signaling [18]. EMSA was performed to check whether heteronemin can inhibit cytarabine-induced nuclear translocation of NF-κB in leukemic cells. As documented in Figure 4C, pre-treatment with heteronemin significantly abrogated cytarabine-induced DNA binding of NF-κB. Next, we studied whether heteronemin affects the cytarabine-induced expression of c-myc, one of the well-studied oncogenes in AML. We observed that cytarabine induced-c-myc over-expression is significantly down-regulated by heteronemin in the cells treated with their combination (Figure 4D).

### Bioinformatic approach to assess and compare the potency of Heteronemin and L-744,832 (Ras FTaseinhibitor) to target Farnesyltransferase

**Figure 5:**
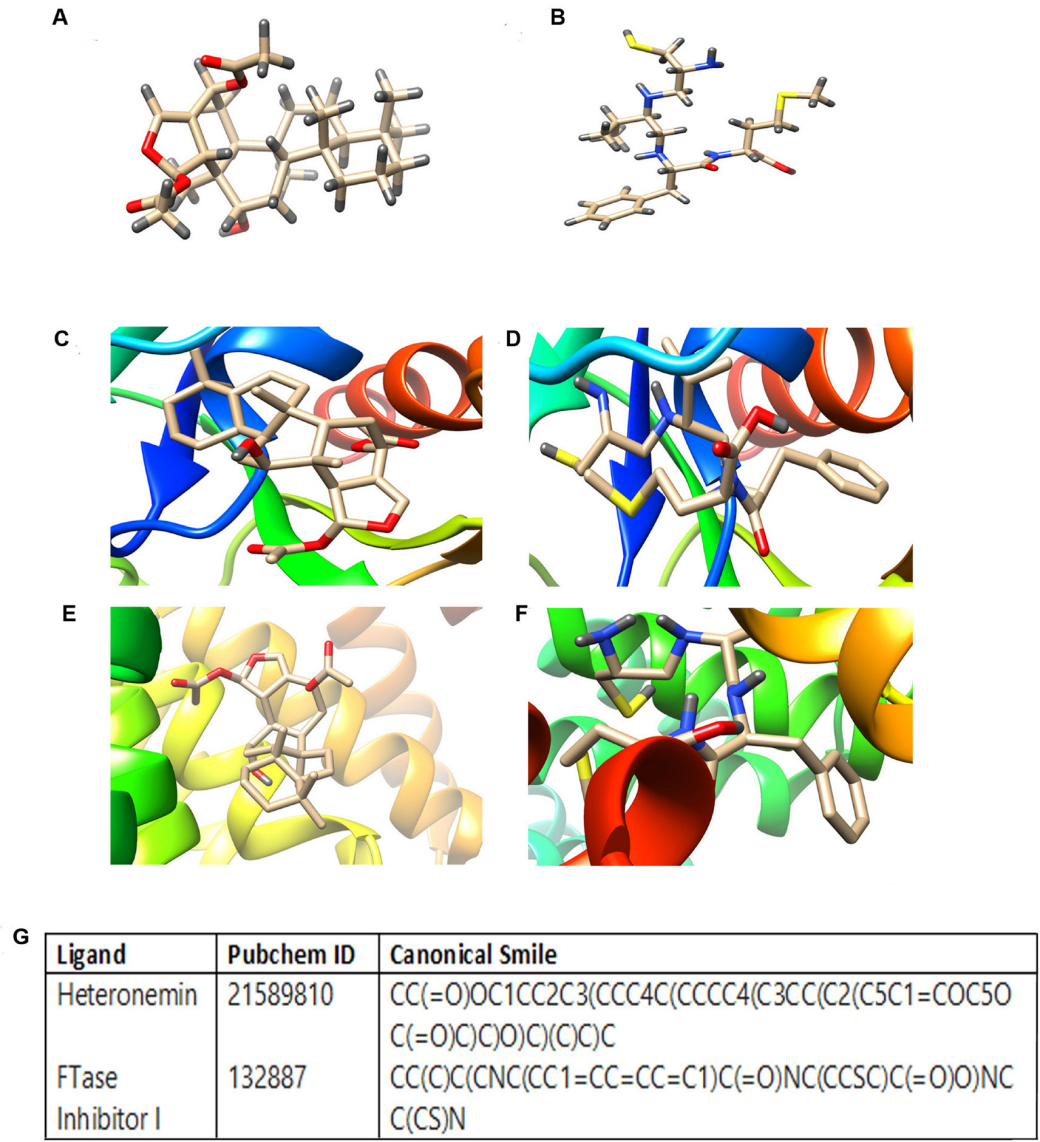
Molecular docking studies showing the binding of heteronemin and FTaseinhibitor1 (L-744,832) in the active site of farnesyl transferase enzyme (**A**) Strucure of Heteronemin (**B**) Structure of FTaseinhibitor1 (**C**) Docked structure of FTalpha - Heteronemin (**D**) Docked structure of FTalpha-Ftaseinhibitor1 (**E**) Docked structure of FTbeta - Heteronemin (**F**) Docked structure of FTbeta-Ftaseinhibitor1 (**G**) PubChem ID and canonical smile of heteronemin and FTaseinhibitor1. Autodock tool of Autodock 4.2.6 package was used to assess the binding efficacy of heteronemin and FTaseinhibitor1 in the active site of farnesyl transferase enzyme.

The number of therapeutic targets have been exponentially elevated upon the completion of the human genome project and the eventual advancement in the techniques to study the structural details of proteins and interaction of protein–ligand complexes with the help of bioinformatics tools have given a new facet to the process of drug discovery. To confirm the role of Heteronemin (Figure 5A) in inhibiting farnesylation of Ras, we adopted a bioinformatics approach to compare the potency of heteronemin and a Ras farnesyl transferase inhibitor in targeting farnesyl transferase. We used Autodock tool of Autodock 4.2.6 package to assess the binding efficiency of ligands to various receptors. FTaseinhibitor1(L-744,832) (Figure 5B) is a commercially available specific inhibitor of Farnesyl transferase. Molecular docking studies were carried out to study whether heteronemin is targeting the chain A or chain B of Farnesyl transferase,. Both chain A and chain B were targeted separately by both ligands and their binding efficiencies were determined. The study revealed that Farnesyl transferase chain A macromolecule forms the binding site comprising of ASN234, TRP237, ASN238, TYR241, PHE242, ASN278, TYR279, GLY282 with heteronemin (Figure 5C) and LEU289, ASP317, GLU320, ASP321, GLU324, TYR355, ILE359, SER362, LEU363, LYS366, HIS367 residues with FTaseinhibitor1 (Figure 5D) and displayed a binding affinity of −6.9 Kcal/mol and −5.9K cal/mol respectively. In the case of Farnesyl transferase chain B, it was observed that this macromolecule forms the binding site comprising of TRP106, ARG202, HIS248, GLY250, TYR251, TYR300, TRP303, TYR361 residues with heteronemin (Figure 5E) and TRP106, TYR154, ARG202, TYR205, CYS206, HIS248, GLY250, TYR251, CYS254, TYR300, TYR361 residues with FTaseinhibitor1 (Figure 5F) and exhibited binding affinity of −7.8 Kcal/mol and −6.2 Kcal/mol respectively. In the case of both chain A and chain B, heteronemin exhibited better binding affinity with Farnesyl transferase than FTaseinhibitor1, thereby proving itself as a superior candidate molecule which can inhibit Farnesyl transferase, thereby preventing Ras activation. Figure 5G shows the PubChem ID and canonical smile of heteronemin and FTaseinhibitor1.

## DISCUSSION

**Figure 6:**
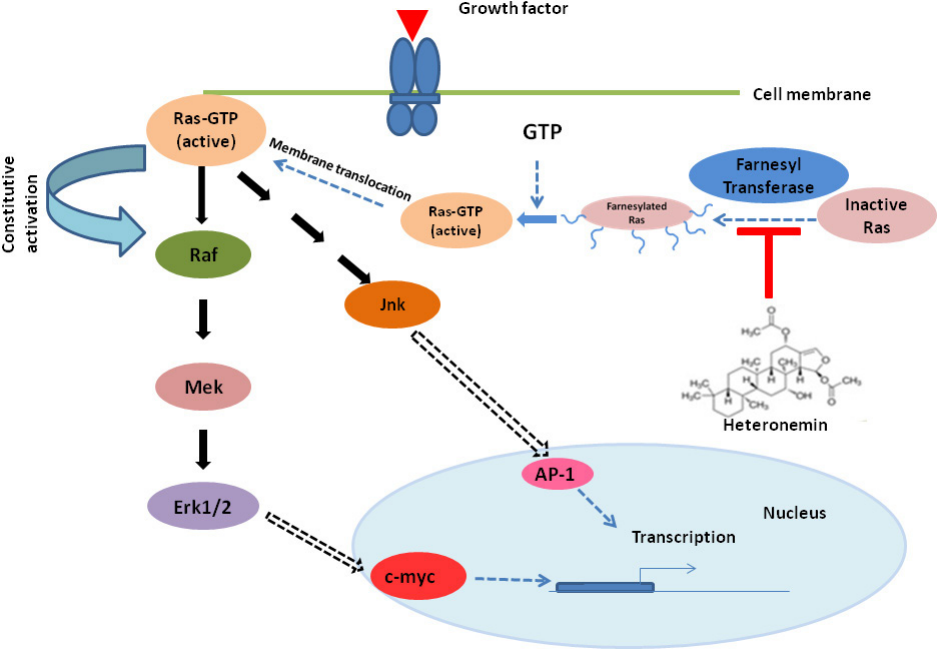
Proposed mechanism of cytarabine sensitization by heteronemin Heteronemin binds to the active site of farnesyl transferase, inhibiting the addition of farnesyl moiety to inactive Ras, thus preventing the translocation of Ras to the plasma membrane from cytoplasm. This in turn prevents the activation of Ras, by inhibiting the formation of Ras-GTP, further inhibiting the MAPK signaling, thus preventing the abnormal proliferation and survival of leukemic cells.

Oncogenic activity of Ras and its association with incidence and progression of AML have been reported earlier by several research groups [19, 20]. Activated oncogenic Ras results in phosphorylation of further downstream signaling molecules including MAPK pathway resulting in their constitutive activation [21]. Several studies have shown that targeting Ras/MAPK pathway exhibits significant anti-leukemic activity [22]. Moreover, several reports attest that, patients who carry activating oncogenic Ras mutations are more sensitive to the cytotoxic effect of cytarabine compared to those having wild type Ras [23–26]. Studies also suggest that side effects such as myelosuppression, neurotoxicity, etc. are associated with high dose cytarabine therapy, making cytarabine a less attractive single agent chemotherapeutic drug [27–29]. Prolonged exposure of leukemic cells to cytarabine causes up-regulation of various survival molecules of MAPK pathway, which play a crucial role in regulating the proliferation of these cells. This will ultimately lead to chemoresistance and relapse of the disease [30, 31]. Chemosensitization is an attractive strategy which makes the cancer cells more susceptible to the action of chemodrugs, by down-regulating the activated survival signals [32]. Here, we report for the first time that a significantly low dose of cytarabine when administered in combination with the marine natural compound, heteronemin, can bring about a synergistic cytotoxic effect and produce a much better anti-leukemic effect, than a higher dose of cytarabine alone. We could clearly observe that the molecular characteristics of apoptosis were significantly enhanced in cells treated with the combination of heteronemin and cytarabine, compared to those treated with the individual compounds. Interestingly, this combination did not affect the viability of PBMCs isolated from the peripheral blood of healthy donors, indicating its biological safety. With the advancement of research and molecular techniques, better understanding of the abnormalities at the molecular level has led to the discovery of various farnesyl transferase inhibitors for AML therapy. There are various reports indicating the effectiveness of farnesyl transferase inhibitors in the treatment of cancers such as leukemia, where Ras is often de-regulated. Many compounds such as tipifarnib and lonafarnib also have entered into clinical trials, upon clearing the *in vitro* studies. However, none have yet been approved by FDA for treatment of AML, probably due to the side-effects associated with them [33]. Being a natural product, we expect heteronemin to be biologically safe, especially at the sub-toxic concentration in which we use it as a chemosensitizer. Moreover, it could effectively prevent cytarabine-induced activation of Ras, as assessed by Ras pull-down assay and inhibited the farnesylation of Ras as illustrated by [^3^H] thymidine incorporation assay. There is no significant difference in the cytotoxicity between HL60 set and HL60+ L-744,832 set for single agent heteronemin or heteronemin+cytarabine since, both L-744,832 and heteronemin targets farnesyl transferase for inhibition. Up-regulation of MAPK signaling which lead to the nuclear translocation and activation of the transcription factor AP-1 is one of the major downstream pathways activated by Ras and is an undesirable consequence often encountered in cytarabine chemotherapy for AML. Heteronemin successfully inhibited cytarabine-induced phosphorylation of all major members of MAPK pathway and in turn inhibited the binding of AP-1 to its respective promoter region in the DNA. The association of NF-κB with progression of malignancy is undisputable and AML is no exception. Constitutive activation of NF-κB has been reported in almost 40% of AML cases [34] and studies indicate that aberrant activation of NF-κB is a crucial event responsible for resistance to cytarabine [35]. Several anti-apoptotic proteins and signaling molecules that offer survival advantages to leukemic cells are transcriptional targets of NF-κB. It has been reported as a potent transcriptional activator of c-myc promoter [36]. c-Myc mRNA contains IRES site (internal ribosomal entry site) that enables translation of c-Myc protein. Ras plays a critical role in the translation of c-Myc through the regulation of IRES translation [37]. Apart from its de-regulation in several myeloid malignancies, over-expression of c-myc has been reported in drug resistance of leukemia [38]. HL60 cells, which we have selected for our current study harbor an amplified c-myc proto-oncogene and the levels of c-myc mRNA are higher in undifferentiated cells which decline rapidly upon differentiation [39]. Heteronemin clearly abrogated the DNA-binding activity of NF-κB and significantly down-regulated the over-expression of c-myc, induced by cytarabine. This is in conjunction with the earlier reports that NF-κB is an activator of c-myc, and when NF-κB activity is abrogated, c-myc expression is negatively affected [40]. Hence, we can delineate that the mechanism of synergism of heteronemin and cytarabine is acting via the farnesylated Ras-MAPK-NF-κB/AP-1 axis (Figure 6). Molecular docking studies also illustrated that heteronemin is having a better binding efficacy towards farnesyl transferase enzyme, than the commercially available inhibitor of the same. We believe that the combination of heteronemin and cytarabine has the potential to become a therapeutic strategy for treating acute myeloid leukemia in the future. However, this warrants for extensive *in vivo* pharmacological studies, followed by clinical trials.

## MATERIALS AND METHODS

### Chemicals

RPMI 1640 medium and fetal bovine serum (FBS) were purchased from Gibco BRL, Grand Island, NY, USA. Antibodies against caspases, phospho-ERK1/2, phospho-JNK and phospho-p38 were obtained from Cell Signaling (Beverly, MA, USA); and that against PARP was purchased from Santa Cruz Biotechnology (Santa Cruz, CA, USA). β-actin, antibody, heteronemin and cytarabine were purchased from Sigma Aldrich (St. Louis, MO, USA). MTT was purchased from (MTT, USB/Amersham Life Science).

### Cell lines

AML cell line HL-60 was gifted by Garcia-Manero, Guillermo (MD Anderson Cancer Centre, Houston, TX, USA). HL60 cells are characterized as acute promyelocytic cells (classification of acute leukemia French-American-British classification (FAB M3)). They carry N-Ras isoform and have c.(del) mutation with a NULL p53 status. The cells are cultured in RPMI-1640 medium supplemented with 10% heat inactivated FBS and antibiotics.

### Isolation of peripheral blood mononuclear cells (PBMCs)

Human peripheral mononuclear blood cells (PBMCs) were isolated from heparin-anti-coagulated blood of healthy human donors after obtaining informed consent from the donors and approval from the institutional human ethics committee of Rajiv Gandhi Centre for Biotechnology (IHEC/01/2017/08) using Histopaque-1077 (Sigma Aldrich, St. Louis, MO, USA) as reported by Ashokan A *et al.* [41].

### Mode of treatment

In all combination treatments, heteronemin (5 nM) was added 6 h prior to cytarabine (1 nM) unless otherwise mentioned. The DMSO concentration in all experiments, including controls, was less than or equal to 0.2%.

### MTT assay

The viability of cells upon treatment with heteronemin and/or cytarabine was determined by MTT assay as described earlier [42] and the relative cell viability percentage is expressed as (Abs_570_ of treated wells/Abs_570_ of untreated wells) × 100.

### [^3^H] Thymidine incorporation assay

[^3^H] Thymidine incorporation assay was performed to assess inhibition of DNA synthesis induced by various compounds as described earlier [32]. Cells (~5 × 10^3^ per well) were treated with required concentrations of the drug after seeding in 96-well plate. After 18 h incubation with the compounds, [^3^H] thymidine was added (0.2 μCi per well) and incubated for 6 h. The cells were washed with PBS; precipitated with 5% trichloroacetic acid and solubilized in 0.2 N NaOH. The relative cell viability was calculated as percentage thymidine incorporation over untreated control.

### Determination of combinatorial effects

Combination index (CI) was determined to analyze whether the compounds are interacting with synergistic or additive effect. It was determined as described by Chou and Talalay [43]. Combinations having CI value <1 were taken as synergistic, those with CI value = 1 were taken as additive and those with CI values >1 were taken as antagonistic. The most effective synergistic combination was selected for further studies.

### Western blot analysis

Total protein isolated from cells after indicated treatments were subjected to Western blotting as described earlier, to analyze the expression status of various proteins in response to individual and combination drugs [32]. Briefly, 40–60 μg of whole cell protein was resolved on a 10–15% polyacrylamide gel. This was then transferred to a PVDF membrane using wet transfer in towbin buffer and the membrane was incubated with the corresponding antibody overnight followed by 3 hr incubation and detected by Enhanced chemiluminescence (ECL) (Millipore, Billerica, MA, USA).

### Annexin-V/PI staining

The extent of apoptosis induced by the compounds was estimated by flow cytometry using Annexin-V apoptosis kit (Santa Cruz, CA, USA) and was performed according to manufacturer's instructions. Analysis was done by flow cytometry using FACS Aria ^TM^, BD Bioscience.

### Preparation of nuclear extracts and EMSA assay

EMSA assay was performed to evaluate DNA-binding activity of NF-κB or AP-1 as described earlier [20]. In brief, 10 μg of nuclear protein was incubated for 30 min with ^32^P-end-labeled double-stranded NF-κB or AP-1 oligonucleotide and the DNA protein complex was resolved in 6.6% non-denaturing polyacrylamide gel, which was dried and visualized by Phosphor Imager (Personal Molecular Imager FX; Bio-Rad Laboratories, Hercules, CA, USA).

### Ras pull-down assay

Ras pull-down assay was performed as per manufacturer's protocol, Ras Pull-down Activation Assay Biochem Kit - (Cat. # BK008-S), Cytoskeleton, Inc.

### Ras farnesyl transferase inhibitor

Ras farnesyltransferase inhibitor, L-744,832, Santa cruz (sc-221800), was added 1 h prior to the pre-treatment of the leukemic cells with the combination of cytarabine and heteronemin.

### Bioinformatic studies

To assess the potency of heteronemin and FTaseinhibitor1 (L-744,832) in targeting Farnesyl transferase (Chain A and Chain B respectively), virtual docking study was carried out at their respective catalytic domains. 3D structure of Farnesyl transferase (Chain A and Chain B respectively) were obtained from RCSB protein database with PDB ID 4Q21, 5P21, 1SA4 and 1BKD with 2.0 Å, 1.35 Å, 2.1 Å and 2.8 Å resolutions respectively. 3D structure of heteronemin and FTaseinhibitor1 were prepared using Frog server from canonical smile obtained from Pubchem with pubchem Id 21589810 and 132887 respectively. Ligands were docked to the receptor chosen for the study using Autodock tool of Autodock 4.2.6 package. Grid map of 40 × 40 × 40 grid point with 0.375 Å spacing were generated using Autogrid program. According to Lamarckian Genetic Algorithm (LGA) with maximum 250000 energy, binding efficiency of ligands to various receptors was determined.

### Statistical analysis

The error bars represent ±S.D. of the experiments. The comparison of mean data among multiple groups was analyzed by ANOVA; ^***^P-values ≤ 0.001, ^**^P-values ≤ 0.01 and ^*^P-values ≤ 0.05; ns represents non-significance.
